# P01-050 – Anakinra in systemic JIA: single center experience

**DOI:** 10.1186/1546-0096-11-S1-A53

**Published:** 2013-11-08

**Authors:** M Pardeo, A Insalaco, C Bracaglia, R Nicolai, AE Tozzi, G Prencipe, F De Benedetti

**Affiliations:** 1 Division of Rheumatology, Department of Pediatric Medicine, Ospedale Pediatrico Bambino Gesù, Rome, Italy

## Introduction

Systemic juvenile idiopathic arthritis (sJIA) accounts for 10-20% of all patients with JIA. The clinical features include fever, evanescent rash, arthralgia and arthritis, myalgia, lymphadenopathy, hepatomegaly, splenomegaly and serositis. Interleukin 1 (IL-1) has been shown to be a major mediator of the inflammatory cascade that underlies sJIA (1). Treatment with anakinra, IL-1 receptor antagonist has been reported to be effective in a subset of patients with sJIA (2).

## Objectives

To assess anakinra as a therapy for sJIA in a single-center series.

## Methods

We reviewed twenty-one consecutive patients with sJIA treated with anakinra for at least 6 months in our institution. The diagnosis of sJIA was established according to the International League of Associations for Rheumatology (ILAR) criteria. We analyzed the effect of Anakinra on fever, rash, number of actives joints, erythrocyte sedimentation rate (ESR), C-reactive protein (CRP), white blood cell count, platelet count and ferritin levels. Clinically inactive disease was defined according to Wallace criteria. Clinical and laboratory data were obtained using a standard data collection form end resulting data were analyzed using Fisher exact test.

## Results

At the beginning of treatment mean age (range) was 8.57 (2.15-16.63) years; 19 of 21 patients had fever and median number of active joints was 3 (1-15). After 6 months of treatment 11 patients (52.3%) met the criteria for inactive disease. Among 21 patients 7 (33%) received anakinra in monotherapy and 14 (66.6%) received anakinra with glucocorticoids. There were no statistically significant differences between the two groups for demographic, clinical and laboratory features*.* Five of 7 pts (71.4%) treated with anakinra alone and 6 of 14 pts (42.9%) treated with anakinra and glucocorticoids met criteria for inactive disease at 6 months (p= 0.361). Among the 21 patients, 10 (47.6%) received anakinra in the first 6 months of disease. There were no statistically significant differences for demographic, clinical and laboratory features among patients who started anakinra in the first 6 months of disease and those that started it after 6 months from onset of disease. At 6 months after initiation of anakinra treatment 8 of 10 patients (80%) who started anakinra during the first 6 months of disease and 4 out of 11 (36.4%) who started anakinra after 6 months of disease reached clinical inactive disease (p=0.08).

## Conclusion

In agreement with several observations, anakinra is effective in a significant proportion of patients with sJIA. Our observation, albeit on a small number, show that association with glucocorticoids does not significantly affect outcome at 6 months and suggest, on the other hand, that earlier treatment may be associated with a better outcome.

## Disclosure of interest

None declared

